# Design and implementation of an evaluation framework for the Epidemic Intelligence from Open Sources (EIOS) system for international public health intelligence at the Robert Koch Institute, Germany, 2023

**DOI:** 10.2807/1560-7917.ES.2026.31.5.2500363

**Published:** 2026-02-05

**Authors:** Mario Martín-Sánchez, Sarah Esquevin, Andreas Jansen, Sofie Gillesberg Lassen

**Affiliations:** 1 Department of Infectious Disease Epidemiology, Robert Koch Institute (RKI), Berlin, Germany; 2 ECDC Fellowship Programme, Field Epidemiology path (EPIET), European Centre for Disease Prevention and Control (ECDC), Stockholm, Sweden; 3 Centre for International Health Protection (ZIG), Robert Koch Institute (RKI), Berlin, Germany

**Keywords:** EIOS, Evaluation, Event-based surveillance, Public Health Surveillance, Pandemic Preparedness

## Abstract

**BACKGROUND:**

Public Health Intelligence (PHI) aims to detect health threats early for a timely and effective response. The PHI team at the Robert Koch Institute (RKI) uses the Epidemic Intelligence from Open Sources (EIOS) system in combination with other sources for detecting signals of international public health threats relevant to Germany. However, while EIOS is increasingly used for PHI worldwide, it is rarely evaluated.

**AIM:**

We designed and conducted an attribute-based evaluation to assess EIOS’s performance for international PHI in 2023 and to identify areas for improvement.

**METHODS:**

We adapted surveillance system attributes and designed attribute-specific data collection methods. We conducted a mixed-method evaluation combining prospective and retrospective operational data collection with feedback from PHI officers.

**RESULTS:**

During 2 weeks in July 2023, the PHI team reported 20 signals: 16 detected using EIOS and four from other sources. Increasing the number of EIOS sources increased timeliness and sensitivity slightly but caused a 35-fold increase in articles to screen (35,546 vs 1,138). The team found EIOS flexible and simple for signal detection but identified challenges in simplicity of signal documenting and reporting and in completeness of EIOS sources screened by the team.

**CONCLUSION:**

The current use of EIOS proved sensitive and timely. However, PHI must balance sensitivity, timeliness and resource requirements. To maintain this balance, we strongly recommend regular evaluations of the use of EIOS for PHI. Our evaluation offers practical guidance for other PHI teams. We recommend integrating EIOS with an event management system to facilitate signal documentation and reporting.

**Key public health message**

**What did you want to address in this study and why?**

The Epidemic Intelligence from Open Sources (EIOS) system collects and processes publicly available information to monitor potential public health threats. At the Robert Koch Institute (RKI), the public health intelligence (PHI) team combines selected EIOS sources with other sources to detect international health threats that could affect Germany. We wanted to evaluate how well EIOS detects public health threats and identify areas for improvement.

**What have we learnt from this study?**

The EIOS system supported timely and sensitive detection of international health threats. The use of a limited number of EIOS sources, complemented with other sources, effectively balanced sensitivity, timeliness and workload. Unsing more EIOS sources marginally increased sensitivity and timeliness but heightened workload i.e. 35 times more articles had to be screened (35,546 for 2 weeks instead of 1,138).

**What are the implications of your findings for public health?**

Balancing sensitivity, timelines and workload through targeted screening can improve public health threat detection without overburdening PHI teams. Regular evaluation of PHI activities using EIOS is essential to achieve this. Our findings provide practical guidance on conducting an evaluation. Nevertheless, further development of evaluation frameworks can improve the use of EIOS for early threat detection and informing public health actions.

## Introduction

Public health intelligence (PHI) is the systematic process of collecting, analysing and interpreting data on potential health threats to timely and effectively inform public health interventions [1,2]. At the German national public health institute, the Robert Koch Institute (RKI), the PHI team detects, verifies, assesses and communicates information on public health events occurring outside Germany that may affect the country following an all-hazards approach (hereafter ‘international PHI’) [3].

Public health intelligence uses information from indicator-based surveillance (IBS) and event-based surveillance (EBS) [4]. An important component of EBS carried by the PHI team is media monitoring, which is conducted using the Epidemic Intelligence from Open Sources (EIOS) system. The EIOS initiative, developed by the World Health Organization (WHO) and European Commission’s Joint Research Centre, is both a community of practice and a system. It is hosted within the WHO Hub for Pandemic and Epidemic Intelligence and led by the EIOS Core Team, which is tasked with further developing and expanding the initiative. At WHO Regional Offices, EIOS focal points serve as support and are the implementing partners of the EIOS initiative at the regional level. The EIOS system is a web-based platform that gathers open-source information including news reports, social media and other online sources [5,6]. It allows the use of filters to sort information articles based on sources, categories (keyword-based classification into diseases, symptoms, outcomes, etc.), geography and language. Epidemic Intelligence from Open Sources system users can create EIOS ‘boards’ to store these filter settings to facilitate the selection and organisation of information for PHI.

The PHI team has used EIOS since its establishment in 2017. In January 2023, the PHI team transitioned to using EIOS as its primary media monitoring system, leading to changes in team working procedures. This transition prompted an evaluation to: (i) determine whether the use of EIOS for international PHI in 2023 was optimising resource use while ensuring timely and sensitive detection of international health threats and (ii) identify potential improvements to current working procedures using EIOS.

Since an EIOS evaluation framework was lacking, we looked at other surveillance system guidelines. Most guidelines for evaluating surveillance systems follow an attribute-based approach, including those from the European Centre for Disease Prevention and Control (ECDC) [7] and the United States (US) Centers for Disease Control and Prevention [8]. Nevertheless, these are usually formulated from an IBS perspective. In this manuscript, we describe the development and application of an attribute-based approach to evaluating EIOS use for international PHI at the RKI.

## Methods

We conducted a mixed-method evaluation combining prospective and retrospective operational data collection with feedback from PHI officers. The new PHI working procedure using EIOS was implemented in January 2023. The evaluation, which ran in parallel with and was incorporated into the PHI working procedures, began in a stepwise manner in June 2023 and was completed in December 2023.

### Sources of information for public health intelligence

In January 2023, the PHI team at the RKI created a customised board within the EIOS system for signal detection (hereafter ‘PHI board’). The PHI board contained articles from 56 sources that had been routinely screened manually by the PHI team before the transition to EIOS. It did not contain any filters based on other criteria (a detailed description of the filters applied is available in Supplementary Table S1). The PHI board was screened once a day in the morning, Monday to Friday, by the PHI officer on duty. In addition, the officer regularly screened other restricted-access sources: Early Warning and Response System (EWRS) of the European Union [9], ECDC daily Round Table Report and weekly threats report [10], information from Global Outbreak Alert and Response Network (GOARN) operational calls [11] and communications through the PHI team mailbox.

### Signal detection, verification, assessment and reporting

The officer on duty performed signal detection against selection criteria adapted from the International Health Regulations (IHR) [12]. Signals were selected if any of five criteria were met: the four criteria from Annex 2 of the IHR ((i) public health impact, (ii) unusual/unexpected event, (iii) risk of international spread and (iv) risk of travel/trade restrictions), plus an additional criterion of high media interest in Germany. After a signal was detected, it was verified by cross-checking with official sources, ensuring authenticity. Once verified, the officer conducted a preliminary assessment of the signal’s relevance to public health in Germany by considering the event characteristics, the occurrence probability, potential impact and national capacity for surveillance and control. The officer presented the signals at the daily round table meeting with all PHI officers (six at the time of the evaluation) and the PHI team lead. The assessment was validated at the round table meeting and the participants made a final decision on reporting. When needed, subject matter experts at the RKI were asked to provide further context for the assessment or additional validation.

Signals approved by the round table (hereafter ‘PHI-relevant’ signals) were included in daily reports and compiled into a weekly report on Fridays. The daily and weekly reports were shared with the Federal Ministry of Health, and the weekly report was also shared with the Federal Ministry for Foreign Affairs and all department heads at the RKI. The following Monday, the PHI team presented the weekly report to the team in charge of the German weekly epidemiological teleconference (EpiLag), composed of members of the Department of Infectious Diseases Epidemiology at the RKI. The EpiLag takes place on Tuesdays (weekly) and is attended by representatives from the Department for Infectious Disease Epidemiology at the RKI, the federal state health authorities and the German armed forces. The EpiLag team selected signals that came from official sources with potential implications for Germany, or were considered unusual, or with attention from media or public health authorities. Subject matter experts at the RKI were consulted to evaluate signal relevance and practical implications for the German Public Health Service [13]. The EpiLag team also reported international signals from other sources if they complied with their reporting criteria.

### Evaluation design

We conducted a mixed-method evaluation integrating quantitative and qualitative methods. Quantitative methods consisted of prospective and retrospective operational data collection and descriptive analysis, while qualitative methods involved gathering feedback from PHI officers to provide explanatory insight into the quantitative results and an adapted nominal group. We structured our evaluation following guidelines from the ECDC Data quality monitoring and surveillance system evaluation handbook [7] and complemented with additional guidance [8,14-18].

We reviewed scientific and grey literature to identify publications on surveillance system evaluation and found 12 relevant attributes for the evaluation. We adapted the identified surveillance system attribute definitions to the EIOS system for international PHI. Subsequently, we identified data collection methods, defined indicators and classified attributes according to evaluation feasibility (low, medium, or high). Feasibility was classified as low when collecting the necessary data for the evaluation would require substantial additional work or major changes to current procedures, medium when data collection was possible but required additional effort or minor adjustments, and high when the data could be collected easily using existing procedures with little to no additional work. Feasibility was assessed based on the evaluation timeline, resources and current PHI working procedures (Table).

**Table:** Surveillance system attributes, their definition, feasibility, data collection method and indicators for evaluating the Epidemic Intelligence from Open Sources system for international public health intelligence at the Robert Koch Institute, Germany, 2023

Surveillance system attribute	Definition	Feasibility	Data collection method	Indicator(s)
**Acceptability**	Willingness of PHI officers at the RKI to perform international PHI using EIOS.	High	**Workshop with the PHI team.** Two questions with answers on a 5-point Likert scale: Question 1: ‘*How willing are you to continue using EIOS for PHI activities?’* (Answers: 1 *‘very unwilling’,* 2 *‘unwilling’,* 3 *‘neutral’,* 4 *‘willing’,* 5 *‘very willing’.*) *Question 2: ‘How likely are you to recommend the use of EIOS to other PHI teams?’* (Answers: 1 *‘very unlikely’,* 2 *‘unlikely’,* 3 *‘neutral’,* 4 *‘likely’,* 5 *‘very likely’.*)	For each question: Number of PHI officers within each category / number of PHI officers. Mean and median.
**Completeness**	Extent to which EIOS contains all necessary sources to perform international PHI at the RKI.	Low	**Prospective EIOS operational data collection and EIOS board source check.** Requires information on: EIOS sources included in the PHI board and their status (active/inactive), EIOS Sources included in the reference board, sources used by the PHI that are not in EIOS.	Number of inactive sources in the PHI board / number of sources in the PHI board. Number of sources from the reference board that could be included in the PHI board. Number of sources not in EIOS used for international PHI.
**Flexibility**	Ability of EIOS to adapt with little additional time or resources to changing information needs for international PHI at RKI (e.g. quickly setting up a new EIOS board or changing filters to monitor one specific event).	High	**Workshop with the PHI team.** Three questions with answers on a 5-point Likert scale: Question 1: *‘How well does EIOS adapt to changing information needs for international PHI?’* (Answers: 1 *‘very poorly’,* 2 *‘poorly’,* 3 *‘moderately well’,* 4 *‘well’,* 5 *‘very well’.*) Question 2: *‘To what extent did this adaptation to the new information needs require additional time and resources?’* (Answers: 1 *‘to a very small extent’,* 2 *‘to a small extent’,* 3 *‘to a moderate extent’,* 4 *‘to a large extent’,* 5 *‘to a very large extent’.*) Question 3*: ‘To what extent do you agree that the time and resources required for the adaptation were reasonable?’* (Answers: 1 *‘strongly disagree’,* 2 *‘disagree’,* 3 *‘neither agree nor disagree*’, 4 *‘agree’,* 5 *‘strongly agree’.*)	For each question: Number of PHI officers within each category / number of PHI officers. Mean and median.
**Positive Predictive Value (PPV)**	Probability of EIOS articles corresponding to PHI-relevant signals.	High	**Prospective EIOS operational data collection.** Requires information on: number of articles screened per board, number of PHI-relevant signals found per board.	Number of PHI-relevant signals per 1,000 articles screened: (number of PHI-relevant signals captured by a board / total number of information articles to be screened in that board) × 1,000
**Sensitivity**	Capacity of EIOS for international PHI at RKI to detect PHI-relevant signals.	Medium	**Prospective EIOS operational data collection.** Requires information on: number of PHI-relevant signals in the PHI board and in the reference board, total number of PHI-relevant signals.	Board sensitivity: number of PHI-relevant signals captured by a board / total number of PHI-relevant signals. Expressed as a ratio and percentage.
**Representativeness**	Extent to which PHI-relevant signals accurately represent international health threats in terms of geographical coverage.	Medium	**Prospective EIOS operational data collection.** For every board, information is required on: number of PHI-relevant signals detected using EIOS for each board stratified by WHO Region and total number of PHI-relevant signals stratified by WHO Region.	For each board, and stratified by WHO region: number of PHI-relevant signals detected using EIOS / total number of PHI-relevant signals.
**Simplicity**	Extent to which the structure and operability of EIOS facilitates the detection, documentation and reporting of PHI signals at RKI.	High	**Workshop with the PHI team.** One statement and one question with responses on a 5-point Likert scale*:* Statement: ‘*Please indicate your level of agreement with the following statement: I think EIOS is easy to use for international PHI at the RKI.’* (Responses: 1 *‘strongly disagree’,* 2 *‘disagree’,* 3 *‘neither agree nor disagree*’, 4 *‘agree’,* 5 *‘strongly agree’.*) Question: *‘To what extent does the use of Epidemic Intelligence from Open Sources for international public health intelligence facilitate each of the following activities: (i) detection, (ii) documentation, (iii) reporting, (iv) knowledge transfer, (v) reproducibility?’* (Answers for each of the five items: 1 *‘to a very small extent’,* 2 *‘to a small extent’,* 3 *‘to a moderate extent’,* 4 *‘to a large extent’,* 5 *‘to a very large extent’.*)	For each question: number of PHI officers within each category / number of PHI officers. Mean and median.
**Stability**	Reliability and ability of EIOS to be operational when needed for international PHI at RKI.	Medium	**Logbook for EIOS technical issues.** Includes date of the problem and characteristics (unscheduled outage or IT problem that makes EIOS operation difficult).	Number of unscheduled outages. Description of EIOS IT problems. Number of days with EIOS IT problems / number of days of use.
**Timeliness**	Ability of international PHI using EIOS at RKI to detect PHI-relevant signals at a time point that allows appropriate public health action.	Medium	**Prospective EIOS operational data collection.** For every relevant signal, information is required on: date of first capture in a PHI source, date of first capture in the PHI board, date of first capture in the reference board, date of first report in a PHI weekly or daily report.	Days between first capture of a PHI-relevant signal in any PHI source and in the PHI board. Days between first capture of a PHI-relevant signal in the PHI board and in the reference board. Days between first capture of a PHI-relevant signal and first report in a daily or weekly report.
**Cost-effectiveness**	Relationship between the timeliness and sensitivity of an EIOS board and the costs needed for its operation.	Medium	**Prospective EIOS operational data collection.** Requires information on: previously calculated timeliness and sensitivity indicators for each board and number of EIOS articles per board. Note: the number of EIOS articles is used as a proxy for the cost as it is related to time invested by staff.	(Number of articles in the reference board – number of articles in the PHI board) / (timeliness reference board – timeliness PHI board). (Number of articles in the reference board – number of articles in the PHI board) / (sensitivity reference board – sensitivity PHI board)
**Usefulness**	Extent to which the use of EIOS for international PHI at RKI leads to public health action.	Medium	**Workshop with the PHI team.** Two statements with responses on a 5-point Likert scale: Statement 1: ‘*Please indicate your level of agreement with the following statement: PHI-relevant signals captured by EIOS lead to public health action*’. (Responses: 1 *‘strongly disagree’,* 2 *‘disagree’,* 3 *‘neither agree nor disagree*’, 4 *‘agree’,* 5 *‘strongly agree’.*) Statement 2: *‘Please indicate your level of agreement with the following statement:* *PHI using EIOS enables early detection of international health threats*’’. (Responses: 1 *‘strongly disagree’,* 2 *‘disagree’,* 3 *‘neither agree nor disagree*’, 4 *‘agree,* 5 *‘strongly agree’.*) **Retrospective data collection on PHI activities.** Requires information on: number of PHI-relevant signals reported in daily reports, number of PHI-relevant signals presented during EpiLag.	For each question during the workshop: Number of PHI officers within each category / number of PHI officers. Number of PHI-relevant signals reported by date of report and WHO-region. Number of PHI-relevant signals presented during EpiLag / total of PHI-relevant signals. Number of PHI-relevant signals presented during EpiLag / total of international signals presented during EpiLag.
**Validity**	Extent to which EIOS articles are free from hoaxes and surveillance artefacts.	Low	**Prospective EIOS operational data collection.** Requires information on: number of EIOS articles that are hoaxes or surveillance artefacts and number of EIOS articles.	Number of EIOS articles that are hoaxes or surveillance artefacts / number of EIOS articles.

EIOS: the Epidemic Intelligence from Open Sources system; EpiLag: the German weekly epidemiological teleconference; PHI: public health intelligence; RKI: Robert Koch Institute; WHO: World Health Organization.

This table was provided to PHI officers as background information to assess the relevance of each surveillance system attribute. The table has been simplified, updated and reworded for publication. Public health intelligence-relevant signals refer to international signals that, after assessment by the PHI round table and possible expert input, were considered relevant to public health in Germany and reported in the daily/weekly report. Further details on the data collection methods column are provided in the manuscript section: Data collection and analysis.

**Figure 1:**
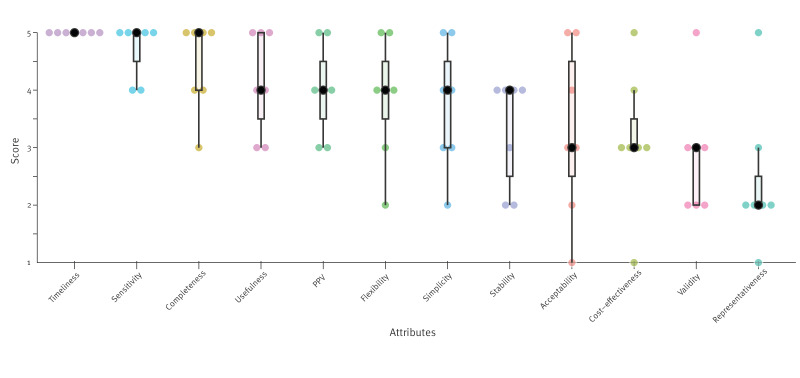
Surveillance system attributes for evaluating the Epidemic Intelligence from Open Sources system for international public health intelligence ranked from high to low median relevance according to current and recent members of the public health intelligence team at the Robert Koch Institute, Germany, 2023 (n = 7 public health intelligence officers)

In June 2023, current and recent PHI officers were asked to rate the relevance of each surveillance system attribute for PHI using the EIOS system at the RKI through a 5-point Likert scale where 1 was *not relevant* and 5 was *very relevant*. Of the eight invited officers, seven participated. We calculated the aggregated rating for each attribute (median, range, and interquartile range). We presented the attribute rating to the PHI team who discussed and validated it as a group. The seven attributes with the highest median relevance (Figure 1) were included in the evaluation: timeliness, sensitivity, completeness, usefulness, positive predictive value (PPV), flexibility and simplicity.

PPV: positive predictive value.

The coloured dots represent each public health intelligence officer’s rating and the black dots the median for each attribute. The boxes extremes show the interquartile range, while the lines extending from the boxes indicate the range of data excluding outliers.

For the seven included attributes, we piloted and implemented the data collection methods between July and December 2023. We collected the data and performed the analysis according to the predefined indicators (Table).

Information from the different data collection methods was gathered into a strengths, weaknesses, opportunities and threats (SWOT) framework. An epidemiologist who was not part of the PHI team but had PHI experience, acted as evaluator and formulated recommendations related to each framework component, which were shared with the team to transform into an action plan.

### Data collection and analysis

#### Retrospective data collection on public health intelligence activities

To evaluate usefulness (Table), in addition to the workshop with the PHI team described below, we analysed information retrospectively over a 6-month period (February to August 2023). We used information from PHI daily reports to describe PHI-relevant signals stratified by signal type (new or update) and WHO Region. Public health intelligence-relevant signals from more than one WHO Region or occurring at the global level were classified as ‘Worldwide’. We identified international signals reported during EpiLag by reviewing the teleconference minutes and comparing them with all PHI-relevant signals.

#### Prospective Epidemic Intelligence from Open Sources operational data collection

**Figure 2:**
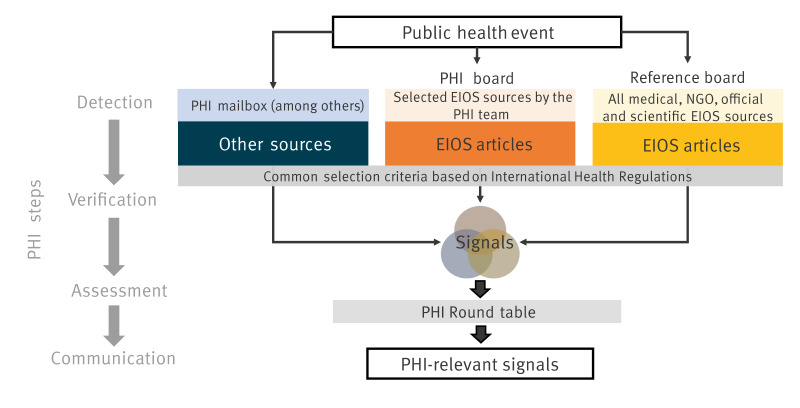
Integration of the prospective operational data collection during a 2-week period of public health intelligence routine activities evaluating the Epidemic Intelligence from Open Sources system for international public health intelligence at the Robert Koch Institute, Germany, 2023

EIOS: Epidemic Intelligence from Open Sources; NGO: non-governmental organisations; PHI: public health intelligence.

To evaluate sensitivity, timeliness and PPV (Table), we conducted prospective data collection over 2 weeks. We designed a reference board including over 1,000 sources labelled as medical, official, scientific and non-governmental organisation (NGO) in EIOS (the filters used to create the reference board are further described in Supplementary Table S1). Between 10–14 and 17–21 July 2023, the PHI officer screened the PHI board and other sources as usual. In parallel, the evaluator screened the reference board, following the same procedure (Figure 2).

We gathered information on all screened EIOS articles using the PHI board and the reference board. To calculate sensitivity and PPV, for each PHI-relevant signal we noted whether it was captured by the PHI board or the reference board on the day of screening. A signal was considered captured by a board on the day of screening if it was part of any article included in that board between the previous screening (the previous morning, except for Mondays) and the current screening, regardless of whether a PHI officer detected it. To calculate timelines, we noted: (i) whether the signal was captured by the PHI board or the reference board at any time, defined as the day of screening or any time within the following 3 months during which a manual follow‑up was conducted to verify whether the signal appeared in any articles on the boards, (ii) the date the signal was first captured by any PHI source and by each of the boards and (iii) the date the signal was reported by the PHI team. An example of the data collection tool is provided in Supplementary Table S2.

The formulas used to calculate these attributes are described in the indicator(s) column in the Table. In addition, for sensitivity, we calculated the 95% confidence interval (CI) for the estimate using the Wilson score method.

#### Workshop with the public health intelligence team

In November 2023, we conducted a 3-hour workshop with current PHI officers (n = 6). The workshop had two components. First, to evaluate simplicity, flexibility and usefulness of EIOS for PHI according to the PHI team, we formulated questions or statements which were scored on a 5-point Likert scale. The Likert scale scores for each question or statement are shown in the Table. PHI officers answered anonymously and the aggregated results were presented with medians and means during the workshop. The findings were then discussed and interpreted within the group. We generated figures based on the workshop results, showing both individual and aggregated outputs, using R version 4.3.0 (http://www.r-project.org).

Second, we conducted an adapted nominal group where PHI officers were asked two questions: (i) ‘*What are the strengths and weaknesses of using EIOS for international PHI at RKI?*’ and (ii) ‘*What would you recommend to improve the use of EIOS for international PHI at RKI?’.* Officers generated ideas individually, wrote them down and then shared their thoughts in a round robin format [19]. This was followed by group discussion and thematic grouping of answers. The results of this adapted nominal group were used to inform the SWOT analysis and the formulation on recommendations.

#### Epidemic Intelligence from Open Sources board source check

To assess completeness, we reviewed the activity of the 56 sources in the PHI board. This review was necessary as no routine monitoring of source activity was available. A source was defined as ‘inactive’ if it was marked as such by EIOS or had no articles indexed in the past 4 months. In addition, we reviewed the sources of all PHI-relevant signals to identify relevant EIOS sources that were not included in the PHI board.

## Results

### Usefulness

**Figure 3:**
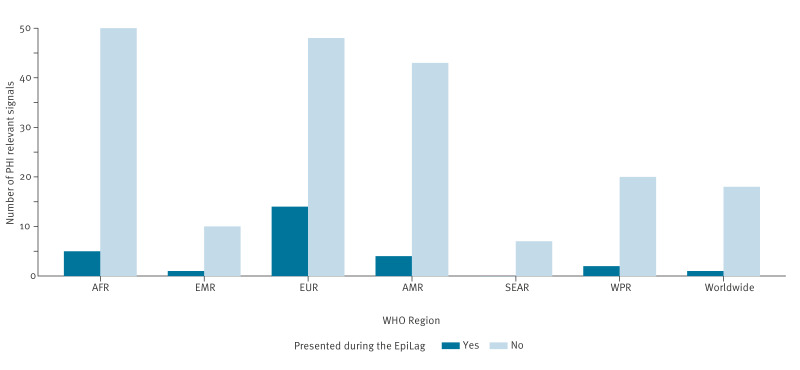
Number of public health intelligence-relevant signals reported over a 6-month period by the public health intelligence team at the Robert Koch Institute stratified by World Health Organization Region and presentation during EpiLag^a^, Germany, February to August 2023

AFR: WHO African Region, EMR: WHO Eastern Mediterranean Region, EUR: WHO European Region, AMR: WHO Region of the Americas, SEAR: WHO South-East Asia Region, WHO: World Health Organization; WPR: WHO Western Pacific Region; Worldwide: signals in more than one WHO region or encompassing all regions.

^a^ EpiLag: the German weekly epidemiological teleconference.

Between February and August 2023, the PHI team reported 223 PHI-relevant signals (108 new events and 115 updates) with a median of 8 per week (interquartile range (IQR): 7–10). Of these, 12% (27/223) were presented during the EpiLag. The proportion of PHI-relevant signals selected for the EpiLag varied by WHO Region (Figure 3). The highest proportion was for signals from the European Region, where 14 of 62 (23%) PHI-relevant signals from that Region were presented. Nine international signals presented in the EpiLag did not come from PHI-relevant signals.

Usefulness was also evaluated during the PHI team workshop. When asked to rate their agreement with the sentence ‘*PHI-relevant signals captured by EIOS lead to public health action*’, two PHI officers disagreed, two neither agreed nor disagreed and two agreed. This resulted in the median: neither agree nor disagree. When asked to rate their agreement with the statement ‘*PHI using EIOS enables early detection of international health threats*’, five agreed and one neither agreed nor disagreed, resulting on the median: agree.

### Sensitivity and positive predictive value

**Figure 4:**
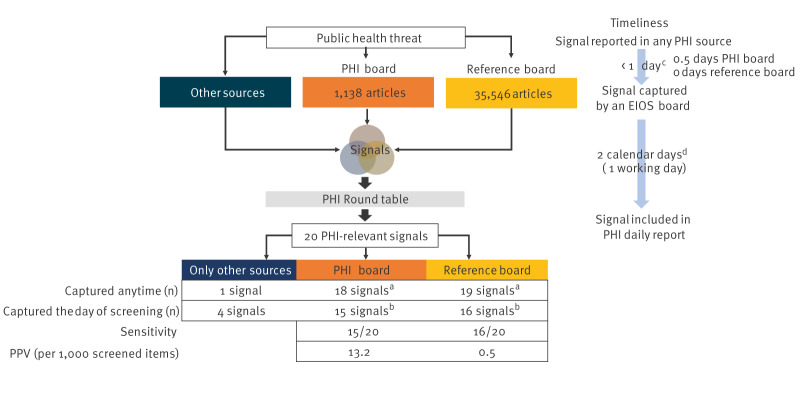
Data collected from the prospective operational data collection and calculation of sensitivity, positive predictive value and timeliness for the evaluation of the Epidemic Intelligence from Open Sources system for international public health intelligence at the Robert Koch Institute, Germany, 2023

PHI: public health intelligence; EIOS; Epidemic Intelligence from Open Sources; PPV: positive predictive value.

^a^18 signals were captured at any time (on the day of screening and up to 3 months afterwards) in both the PHI board and the reference board.

^b^15 signals were captured on the day of screening in both in the PHI board and the reference board.

^c^ Median days between time points for the 19 signals captured at any time by either board.

^d^ Median days between time points for the 16 signals captured on the day of screening by either board.^..^

During the prospective data collection, 1,138 articles from the PHI board and 35,546 from the reference board were screened by the PHI officer and the evaluator, respectively. Twenty signals were reported in daily PHI-reports during the 2 evaluation weeks. Of these, 16 were captured by the reference board (sensitivity: 16/20; 80%, 95% CI: 58–92%), and 15 by the PHI board (sensitivity: 15/20; 75%, 95% CI: 53–88%) on the day of screening. The PPV was 0.5 per 1,000 screened articles for the reference board and 13.2 per 1,000 screened articles for the PHI board. The four signals not found in EIOS came from restricted official sources (n = 2 from EWRS, n = 1 from the PHI-team mailbox and n = 1 from the ECDC daily threat report) (Figure 4). Three signals in the reference board and four in the PHI board were captured after the day of screening and hence not included in the sensitivity and PPV calculations.

### Timeliness

For the 19 PHI-relevant signals captured by the boards at any time, PHI-relevant signals were captured by the reference board a median of 0.5 days (IQR: 0–1.75) earlier than by the PHI board. Eleven were captured by the PHI board on the same day, five the following day, two after 2 days and one was not captured by the PHI board.

For the 16 signals captured the day of screening using either board, the median time between capture and inclusion in the PHI report was 2 days (IQR: 1–3). After excluding non-duty days in the time difference, the median was 1 day (IQR: 1–2) (Figure 4).

### Simplicity

**Figure 5:**
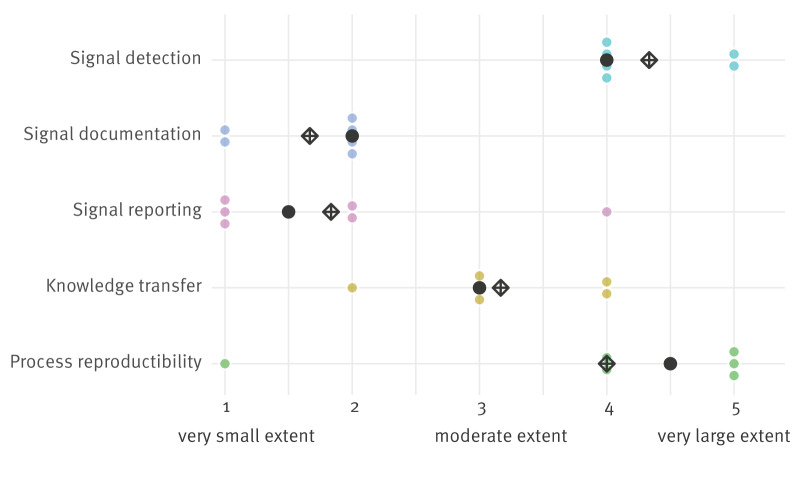
Answers to the question ‘To what extent does the use of Epidemic Intelligence from Open Sources for international public health intelligence facilitate each of the following activities?’ from public health intelligence officers at the Robert Koch Institute, Germany, 2023 (n = 6 public health intelligence officers)

When asked for agreement about the statement ‘*I think EIOS is easy to use for international PHI at the RKI*’ the median was 4 of 5 (the equivalent to ‘agree’). The extent to which EIOS facilitated tasks varied. The most facilitated tasks were process reproducibility (median: 4.5, between ‘to a large extent’ and ‘to a very large extent’) and signal detection (median: 4, ‘to a large extent’). The least facilitated tasks were reporting (median: 1.5, between ‘to a very small extent’ and ‘to a small extent’), information documentation (median: 2, ‘to a small extent’), and knowledge transfer between PHI officers (median: 3, ‘to a moderate extent’) (Figure 5).

The coloured dots represent each officer’s rating, the black dots the median and the diamonds the mean for each activity.

### Flexibility

In response to the question, ‘*How well does EIOS adapt to changing information needs for international PHI?’,* the median answer was 4 (‘well’). When asked to rate *‘To what extent did this adaptation to the new information needs require additional time and resources?’*, the median answer was 3 (‘to a moderate extent’). With a median answer of 4.5 (‘agree’ to ‘strongly agree’), to the question ‘*To what extent do you agree that the time and resources required for the adaptation were reasonable?’* officers agreed that the time and resources needed for adaptation were reasonable.

### Completeness

Of the 56 sources from the PHI board, 35 (63%) were active in December 2023, 16 did not have any article indexed before or since July 2023, although they were not inactive according to EIOS, and five were inactive according to EIOS. Seven sources from the reference board were identified for future inclusion in the PHI board based on sensitivity and timeliness results.

### Summary of the strengths, weaknesses, opportunities and threats analysis

Identified strengths included the simple and user-friendly EIOS interface, high flexibility and reproducibility, and the EIOS web-based open source system supported by a large community and the WHO. Identified limitations included the difficulty of using EIOS for documenting and reporting signals, the high number of inactive sources leading to a low completeness of the PHI board, the variable perception of usefulness and some user experience issues identified by PHI officers.

Opportunities to improve EIOS for PHI included integration with event management systems and other technical improvements (such as further customisation of information export options), expansion of the EIOS community of practice and ongoing evaluation of EIOS performance to improve the system and PHI workflows. However, threats such as resource constraints, rapid technological changes that may render the system obsolete if not incorporated in a timely manner, reliance on external sources and cybersecurity risks could affect the use of EIOS for PHI. This SWOT analysis informed the formulation of recommendations for the PHI team, including revising the PHI board, conducting continuous and periodic evaluation of EIOS and PHI activities, establishing contingency plans to anticipate future challenges and creating a systematic approach to gather feedback and share it with the EIOS core team for EIOS improvement. The detailed recommendations and SWOT are shown in Supplementary Table S3 and Supplementary Table S4.

## Discussion

We designed and implemented an evaluation framework to assess and optimise the use of EIOS at the RKI for the sensitive and timely detection of international health threats to Germany. Overall, the current use of EIOS was timely and sensitive, and required screening only a limited number of articles. However, areas for improvement were detected. These included the high number of inactive sources routinely used by the PHI team, highlighting the need for regular evaluations, as well as challenges in the use of EIOS for facilitating documenting and reporting signals, which could be addressed through integration with an event management system.

During the evaluation of the EIOS system, the PHI team used a customised EIOS board with 56 sources, which detected 15 of 20 reported PHI-relevant signals. Approximately 76 articles had to be screened to detect one signal. Using a higher number of EIOS sources would have detected one more signal and improved timeliness by half a day, but would have required screening 35,546 articles instead of 1,138. Four signals originated from sources outside EIOS that are not publicly available and will still need to be screened in parallel with EIOS.

Regarding usefulness, over 200 PHI-relevant signals were reported during 6 months. Nevertheless, our definition relating usefulness to public health actions was subject to discussion. Only two of six officers agreed that ‘*PHI-relevant signals captured by EIOS lead to public health action*’, with a different understanding of ‘public health action’ emerging when interpretating results. For some officers, communicating signals to relevant stakeholders was considered an action, while for others, action was what recipients did with the received information. Although this aspect was beyond the scope of our evaluation, it highlighted a previously described gap in surveillance, which is also applicable to PHI: the link between dissemination of output and public health actions [20]. Further evaluation is necessary to determine how and to what extent PHI activities using EIOS may impact public health.

Simplicity has previously been assessed for PHI systems, but typically focussed on the overall system rather than on specific processes [21]. In our evaluation we assessed simplicity for specific PHI steps. We found that EIOS was considered simple for signal detection but not for documenting and reporting. Public health intelligence officers noted that quantifying and following up on signals detected through EIOS was not easy, and that transferring information from EIOS into the daily reports remained time consuming. The PHI officers proposed targeted improvements, including more customisable export options or better integration of EIOS with event management systems, which are important tools used routinely in PHI work [22,23].

Countries within all WHO Regions, WHO regional offices and other health agencies have integrated EIOS into their PHI activities [1,6,24,25]. The EIOS system also played a major role in the WHO’s COVID-19 pandemic PHI activities [26] and EIOS has been used to complement existing surveillance during mass gatherings [27,28]. Nevertheless, EIOS evaluations are rare. Published evaluations include an assessment of EIOS's capacity to strengthen data collection and mapping for neglected diseases, an assessment of EIOS's performance in detecting influenza outbreaks and an evaluation of EIOS in the WHO African Region showing its performance in the early detection of public health events [29-31]. The EIOS system was also used to evaluate the sensitivity of the World Organisation for Animal Health’s World Animal Health Information System using media reports [32]. Comprehensive attribute-based evaluations of the potential of EIOS for PHI were not identified in published scientific literature.

Establishing evaluation guidelines for teams using EIOS could motivate further evaluations, facilitating continuous improvement of activities and processes, and increase the comparability of results between different PHI teams. We developed our evaluation framework based on existing guidelines, mainly derived from IBS. It is possible that a specific framework for EBS or PHI would have been more appropriate for our evaluation. However, there is a lack of standardisation in the monitoring and evaluation of EBS systems and evaluation guidance is scarce. This is likely due to the diversity of EBS processes and systems, as well as the difficulty of dealing with unstructured information compared with IBS [33,34]. In 2024, a manuscript outlining a new framework that provides a structured approach to the monitoring and evaluation of EBS activities was published, addressing a significant gap in the field [34].

Our evaluation has several limitations. First, during the workshop, some of the questions were interpreted differently by the different PHI officers, particularly the questions related to usefulness. This could explain the variability in certain responses, but it also served to raise important points for discussion. Second, there is no gold standard of PHI activities with which we can compare our results. For example, establishing a denominator for sensitivity is challenging as signals reported by others may not be considered relevant for the PHI team, given the different context and mandates. In our study we used the total number of reported signals as a denominator to compare the performance of the PHI and reference EIOS boards. However, this denominator does not ensure that all relevant signals, e.g. those not captured by the sources screened by the team, are detected and it is likely that the sensitivity may have been lower if a different denominator had been chosen. Third, despite selection criteria and the round table, PHI-relevant signal detection sometimes depends on the person on duty. This individual variability may have influenced the number of signals reported during the evaluation and affected reproducibility. Last, we focused exclusively on the boards used by the PHI team at the RKI, which were set up independently from the EIOS core team and the regional focal point. The boards rely on filtering based on sources rather than categories. This approach is dependent on the labelling of EIOS sources and limits the generalisability and comparability of our findings to other PHI teams using boards based on categories. The expertise from the EIOS core team and regional focal points should be incorporated when creating up boards and conducting evaluations.

## Conclusions

Given the growing importance of PHI, this evaluation offers practical insights that can help establish good practices for other PHI teams and the global EIOS community of practice. We recommend PHI teams using EIOS to assess their board performance, particularly in terms of sensitivity, timelines and PPV. Even small gains in sensitivity may come at the cost of a sharp drop in PPV, increasing the number of articles to be screened. Tailoring boards to PHI objectives and regularly monitoring PPV are essential for ensuring efficiency in PHI work. For those starting to use EIOS or considering it, PHI officers at the RKI concluded that, despite facilitating signal detection, it might not ease the burden related to documenting and reporting. Integrating EIOS with an event management system could help address this issue. Further evaluations and research on the use of EIOS for PHI are needed, particularly to assess its usefulness and to generate insightful feedback for system improvements.

## Data Availability

The data that support the findings of the evaluation are available from the corresponding author upon reasonable request.

## Note

The Robert Koch Institute has a memorandum of understanding and ongoing collaboration with the WHO Hub for Pandemic and Epidemic Intelligence (WHO Hub in Berlin), which hosts the EIOS initiative. The evaluation and manuscript writing were conducted independently of the joint projects between the RKI and the WHO Hub in Berlin and no funding was received from them the WHO Hub in Berlin for this project.

## Supplementary Data

326000 Supplementary Material
