# Supplementary material for: Design and implementation of an evaluation framework for the Epidemic Intelligence from Open Sources (EIOS) system for international public health intelligence at the Robert Koch Institute, Germany, 2023
Source: Euro Surveill. 2026 Feb 5;31(5):2500363. doi: 10.2807/1560-7917.ES.2026.31.5.2500363 (PMC12881845; doi:10.2807/1560-7917.ES.2026.31.5.2500363)
Supplement: Supplementary Material [file 25-00363_MARTIN-SANCHEZ_Supplement.pdf]

This supplementary material is hosted by *Eurosurveillance* as supporting information alongside the article “Design and implementation of an evaluation framework for the Epidemic Intelligence from Open Sources (EIOS) system for international public health intelligence at the Robert Koch Institute, Germany, 2023” on behalf of the authors, who remain responsible for the accuracy and appropriateness of the content. The same standards for ethics, copyright, attributions and permissions as for the article apply. Supplements are not edited by *Eurosurveillance* and the journal is not responsible for the maintenance of any links or email addresses provided therein.

Supplementary Table S1. Description of the PHI board and the reference board for the evaluation of EIOS for international public health intelligence at the Robert Koch Institute, Germany.

	PHI board	Reference board
**Filters based on categories**	No filters applied.	No filters applied.
**Filters based on source subjects**	No filters applied.	Yes. Selected source subjects: medical, medical official, NGO, Official, Science.
**Filters based on source types**	No filters applied.	Yes. Selected source types: Medical, TVRadio, social media, web news, wire.
**Filters based on specific sources**	Yes. Selected sources based on previous experience of the PHI Team at the RKI.[a]	No filters applied.
**Number of sources**	56	>1,000

[a] Selected sources in the PHI board as 1 January 2023: ECDCnews, Facebook-ECDC.EU, Facebook-WHO, Twitter-CIDRAP, Twitter-DrTedros, Twitter-ECDC_EU, Twitter-ECDC_Flu, Twitter-ECDC_Outbreaks, Twitter-ECDC_VPD, Twitter-FluTrackers, Twitter-HelenBranswell, Twitter-OWMorgan, Twitter-ProMED_EAFR, Twitter-Reuters_Health, Twitter-WHO, WHODON, africacdc, bag, bmg, cdc-media, cidrap, cidrap-mail, ecdc, eurosurveillance, eurosurveillance-add, flutrackers, goeg, outbreaknewstoday, polioeradication, promed, promed-ahead, promed-amr, promed-eafr, promed-edr, promed-esp, promed-fra, promed-mail, promed-mbd, promed-mbds, promed-port, promed-rus, promed-soas, promedmail.org-38, reuters, statnews, who, who-afro, who-ebola, who-emro, who-emro-html, who-europe, who-regions, who-ru, who-searo, who-wpro, who.bulletin.

Supplementary Table S2. Example of the data collection form for the prospective operational data collection for the evaluation of EIOS for international public health intelligence at the Robert Koch Institute, Germany.

Data collection form implemented in an Excel sheet with drop-down lists and a dedicated column for each signal.

	Signal_X
**Disease, Country**	[Free text]
**Type of signal**	*New event / update*
**WHO region**	*AFR / EMR / EUR / AMR / SEAR/ WPR / Worldwide*
**Date of screening**	[Date]
**Relevance assessment**	*Very low / low / medium / high*
**Source**	
Selected by PHI officer (PHI board)	*Yes/No/NA*
Captured in PHI board the day of screening	*Yes/No/NA*
Selected by evaluator (reference board)	*Yes/No/NA*
Captured in reference board the day of screening	*Yes/No/NA*
Source (if not an EIOS signal)	[Free text]
Original source (as in report)	[Free text]
Comments on source	[Free text]
**Timeline**	
**Date of event onset**[a]	[Date]
**Captured in PHI board at any time**	*Yes/No/NA*
**Captured in reference board at any time**	*Yes/No/NA*
**Date of first capture (PHI sources)**	[Date]
**Date of first capture in EIOS (any board)**	[Date]
**Date of first capture in PHI board**	[Date]
**Date of first capture in reference board**	[Date]
**Date of first reported by PHI team**	[Date]
Comments on timeline	[Free text]

[a]Information collected in an exploratory manner. For outbreaks or single cases of relevant diseases, we consider the onset of symptoms of the first case and if not available, the date of diagnosis. For unexpected increases in disease incidence, we used the date of the change in trend, if reported in the information article.

Supplementary Table S3. SWOT analysis from the evaluation of EIOS for international public health intelligence at the Robert Koch Institute, Germany.

The information from the attribute-based evaluation and the qualitative evaluation by public health intelligence (PHI) officers (including the discussion of the results of the attribute-based evaluation and the answer to the questions: "*What are the strengths and weaknesses of using EIOS for international PHI at the Robert Koch Institute (RKI)?*" and "*What would you recommend to improve the use of EIOS for international PHI at RKI?"*) have been integrated following a SWOT framework.

INTERNAL FACTORS

STRENGTHS	WEAKNESSES
S1. Good balance between sensitivity and positive predictive value of the PHI board. The use of a relatively low number of sources in the PHI board was sensitive enough for the aims of the PHI team at RKI without supposing an excessive workload in terms of articles to be screened.<br><br>S2. Simplicity and ease of use for international PHI. The EIOS system is user-friendly, with most officers finding it easy to learn and use for international PHI.<br><br>S3. Flexibility. EIOS is adaptable to changing information needs with reasonable additional time and resources required.<br><br>S4. Process reproducibility. The use of EIOS aids in enhancing the reproducibility of the PHI processes.<br><br>S5. Web-based and Open Source. Easy accessibility, free of charge, and potential for community-driven improvements.<br><br>S6. EIOS community and support from WHO (EIOS core team). Provides a great opportunity for collaboration and knowledge sharing.	W1. Limited use for documentation and reporting. It is less effective in facilitating information documentation and reporting of signals.<br><br>W2. Low completeness in terms of sources of the PHI board at RKI, considering the high number of sources that were not active approximately one year after its creation.<br><br>W3. Manual work required. Still requires significant manual effort to translate information items into PHI reports.<br><br>W4. Varying perceptions of usefulness. Not all team members agree on the extent to which EIOS for PHI leads to public health action.<br><br>W5. User experience issues. Bugs and interface issues that interfere with the EIOS user experience.<br><br>W6. Ambiguities in categories and feedback use. Perception of a lack of transparency in category definitions by some PHI officers and in how user feedback is utilized by WHO (EIOS core team).

EXTERNAL FACTORS

OPPORTUNITIES	THREATS
O1. Integration of EIOS with other systems for international PHI at RKI and other technical improvements. Potential for integration with event management systems (EMS) for improved efficiency in the PHI work or further customization of information export options to facilitate the reporting.<br><br>O2. Expansion of the EIOS community. Leveraging the PHI team, EIOS community, and WHO support for further development and exchange.<br><br>O3. Continuous Improvement and Evaluation of EIOS and PHI activities. Regular evaluation and feedback mechanisms can contribute to the improvement of the EIOS initiative and the PHI working procedures.<br><br>O4. New source inclusions in the EIOS PHI board. Consideration of additional sources based on the results of the evaluation to enhance sensitivity, timeliness, and completeness of the conventional board.	T1. Resource constraints. Additional resources may be required for workflow adaptation in case of changing information needs or further integration of evaluation in the routine work.<br><br>T2. Rapid technological changes. If advances in technology and data analytics (such as artificial intelligence) are not incorporated into EIOS in a timely manner, there is a risk that PHI working practices will become obsolete.<br><br>T3. Dependence on External Data Sources. Current PHI board relies on secondary sources and hence, on their quality and timeliness.<br><br>T4. Technical malfunctioning and cybersecurity Risks. EIOS as a web-based platform, there are inherent risks related to stability and data security.

Supplementary Table S4. Recommendations from the evaluation of EIOS for international public health intelligence at the Robert Koch Institute, Germany.

SWOT mapping	Recommendation	Proposed action-points	Proposed timeline
S1-5<br>W2<br>O3-4<br>T3	Revise and improve the PHI board to increase completeness while ensuring high sensitivity, positive timeliness, and timeliness.	▪ Keep overall working procedures and practices, including the use of EIOS as main system for PHI at RKI.<br>▪ Delete current sources from the conventional board that are no longer active.<br>▪ Include new sources to the conventional board based on evaluation or previous experience ensuring board resilience in case of further loss of EIOS sources.<br>▪ Assess whether the changes are likely to increase the workload and reassess the included sources accordingly.	Q1 2024
S4<br>W4<br>O1,3-4<br>T2-T3	Enhance the incorporation of evaluations within the operational framework and SOPs of the PHI Team.	▪ Continuous and periodic evaluation of the use of EIOS for international PHI at RKI based on the resources available for that at the PHI team.<br>▪ Revise routinely if sources included in the conventional board are active, and if so, evaluate them routinely. Keep track of sources included in the conventional board over time.<br>▪ Consider an evaluation of the usefulness of the PHI activities involving stakeholders from inside and outside RKI (for example recipients of PHI weekly and daily reports)	Q1-Q2 2024
S3<br>W5<br>O4<br>T1-4	Develop contingency plans to anticipate and address unforeseen challenges, ensuring robustness in response to potential disruptions.	▪ Create a contingency plan containing online sources outside EIOS and procedures that could be used in days or time periods in which the EIOS system is not operative (short-term and long-term back up plan).<br>▪ Create a list of EIOS sources that could be included in the conventional board (or a backup conventional board) in case of necessity or malfunctioning of current EIOS sources in the conventional board.	Q1-Q2 2024
S6<br>W1,3,5-6<br>O1,2<br>T4	Implement a systematic approach to streamline communication channels and feedback mechanisms in collaboration with the EIOS Team for enhanced coherence and efficiency.	▪ Organise and facilitate routine discussions among the PHI team members regarding the functionalities of EIOS, encompassing its communication and collaboration features.<br>▪ Develop a structured system/shared document to collect regular problems and bugs detected during the routine work with EIOS.<br>▪ Agree on bilateral feedback mechanisms with the EIOS Core Team regarding EIOS problems, bugs and features.<br>▪ Explore forming a collaborative working group with the EIOS Core Team to seamlessly integrate and develop an event management system, facilitating signal reporting.	Q2 2024
